# Something to Interest Each Reader

**Published:** 1897-06

**Authors:** 


### SOMETHING TO INTEREST EACH READER.

WE learn of the abandonment, by one of the veterinarians of western Pennsylvania, of the proprietary-medicine scheme which he adopted as the "Mulberry Seller's" plan to get rich quick.

Parasites of the stomach are causing heavy loss among sheep at Lagrange, Ind.

Breeders of dark-colored hogs are having a constant fight to offset the tendency to white or light-colored markings in the offspring.

The shipment of sheep affected with scab from one State to another through the West is giving the Bureau of Animal Industry much concern.

Fifteen thousand dollars was the price paid for the famous horse "Joe Patchen" at the auction-sale in Chicago.

Live steam is one of the best methods of ridding barns of mites and lice, and is equally beneficial in chicken-houses.

At the sale of trotters in Chicago, May 4th to 6th, some ten horses were bought by foreign buyers. Surely the trotter is winning his way abroad as well as at home.

Veterinarian G. A. Banham, of England, suggests the formation of an insurance association among veterinarians for the purpose of partially indemnifying owners of animals where the latter are injured, develop tetanus, blood-poisoning, have open-joints, etc., incidental to operations or while under treatment by veterinarians.

A lady-student has qualified for her first professional examination at the Royal Veterinary College, London, England.

The introduction of foxes into Australia for sport, and with the hope that they might help to eliminate the rabbit-pest, has brought another infliction upon the stock-raisers. Many sheep's tongues are lacerated so badly by the foxes that the sheep die.

Prof. Motley Axe, of Great Britain, is quoted as authority for the statement that one in five of the milk-cows of the United Kingdom are tuberculous, and if slaughtered and paid for at a valuation of £4 per head, it would take £11,000,000 to reimburse the owners.

The young stallion Orme, son of Ormonde, is thought to owe his recent display of high temper and unmanagebleness to a diseased tooth.

Veterinarian John Minchin, of Goshen, N. Y., is the possessor of the bridle-bit of the famous imported thoroughbred stallion, Messenger.

Dr. Robert Koch, the eminent German bacteriologist, who has gone to South Africa to make a study of the rinderpest, is doing admirable work in this line. His investigations have already convinced him that this malady can be conquered by a resort to the same means which have robbed diphtheria of its terrors. Dr. Koch reports that he can now render healthy cattle immune from attack, and has had some success in curing those upon which the disease, hitherto regarded as invariably fatal, had fastened itself. "The work already done," he writes, "convinces me that the rinderpest can be eradicated with little difficulty and within a comparatively short time."

*Proposed A. J. C. C. Scale of Points—Cows and Heifers:*

1. Head lean and not large, face dished, broad between the eyes, wide full muzzle, eyes full and bright, clear horns, counts 6.

2. Neck not too thick, rather long, with clean throat, good withers and shoulders, counts 6.

3. Back good length, level to setting on tail, tail small in bone and reaching below hock, good switch, counts 5.

4. Hips well apart, rump long and not sloping, broad across loins, counts 8.

5. Barrel long, well rounded out, ribs well apart, deep at flank, counts 14.

6. Hide soft and flexible, of reasonable substance, color of inside of legs at thigh and armpit and inside of ear, tinted yellow, counts 7.

7. Legs short and well apart, hind legs comparatively straight and thin at thighs, counts 7.

8. Udder well proportioned, capacious teats, good sized and well placed, milk veins hard and full, extending well forward, counts 32.

9. Disposition, general appearance, and apparent constitution, counts 15.

*Bulls:* The same scale of points shall be used in judging bulls, omitting No. 8. Add 20 points for well-placed teats, and thin in thigh; due allowance should be made for masculinity, but when bulls are exhibited with their progeny in a separate class add 20 points for progeny.

Pure-bred pigs weigh at birth from $2\frac{1}{4}$ to $2\frac{3}{4}$ pounds; at a week old, nearly double this weight.

Among the latest inscriptions on a Western veterinarian's bill-heads we note the following: "All bills due when services are rendered; cash fully appreciated; collections made monthly; even-handed justice to all, but no miracles performed."

In a Maine family of eight children, using the milk of a cow suffering from tuberculosis, three children have recently died of tubercular pneumonia, and three others are reported beyond the hope of recovery. The other two are ill. Another child of the household refusing the milk is in good health.

The returns from the Department of Agriculture show that horses have decreased 5 per cent. from the previous year and mules 2 per cent. Their numbers are increasing in the South and on the South Atlantic coast, but are decreasing elsewhere.

Spain has purchased for her Cuban army some three thousand horses, mostly from Texas.

